# Supplementary material for: Urine-Derived Stem Cells: Applications in Regenerative and Predictive Medicine
Source: Cells. 2020 Feb 28;9(3):573. doi: 10.3390/cells9030573 (PMC7140531; doi:10.3390/cells9030573)
Supplement: Supplementary file 1 [file cells-09-00573-s001.pdf]

SUPPLEMENTAL MATERIAL

Urine-derived Stem Cells: Applications in Regenerative and Predictive Medicine

Guida Bento (1), Aygul K. Shafigullina (2), Albert A. Rizvanov (2,3), Vilma A. Sardão (1), Maria Paula Macedo (4, 5,6), Paulo J. Oliveira (1)*

(1) CNC – Center for Neuroscience and Cell Biology, UC-Biotech, University of Coimbra, Cantanhede, 3030-789, Portugal

(2) Institute of Fundamental Medicine and Biology, Kazan Federal University, Kazan, 420008, Russia

(3) Faculty of Medicine and Health Sciences, University of Nottingham, LE12 5RD, UK

(4) Centro de Estudos de Doenças Crónicas (CEDOC), NOVA Medical School-FCM, Universidade Nova de Lisboa, Lisboa, 1169-056, Portugal

(5) APDP-Diabetes Portugal Education and Research Center (APDP-ERC), Lisboa, 1250-189, Portugal

(6) Departmento de Ciências Médicas, Instituto de Biomedicina - iBiMED, Universidade de Aveiro, Aveiro, 3810-193, Portugal

* Corresponding Author
Paulo J. Oliveira, Ph.D.
CNC – Center for Neuroscience and Cell Biology, UC Biotech, University of Coimbra, 3060-197 Cantanhede, PORTUGAL
phone: +351-231-249-195, fax: +351-231-249-179
email: pauloliv@cnc.uc.pt

Supplemental text: Culture conditions, biological characteristics and differentiation of UDSC

Culture medium composition, presence of serum and growth factors and variation in salt concentration influence cell morphology, phenotype and growth capabilities. When UDSC were described for the first time, Zhang et al. cultivated those cells in a medium for urothelial cells [1,2]. Composition of this medium was in 1:1 ratio keratinocyte-serum free medium (KSFM) (supplemented with 5 ng/mL EGF, 50 ng/mL bovine pituitary extract, 30 ng/mL cholera toxin, 100 U/mL penicillin and 1 mg/mL streptomycin) and progenitor cell medium (supplemented with 0.75 DMEM, 0.25 Hamm's F12, 10% FBS, 0.4 g/mL hydrocortisone, 10^{-10} M cholera toxin, 5 ng/mL insulin, 1.8×10^{-4} M adenine, 5 g/mL transferrin plus 2×10^{-9} M

3,39,5-triiodo-L-thyronine, 10 ng/mL EGF and 1% penicillin-streptomycin). Later, Bhadawaraj et al. [3] and Liu et al. [4] proposed other medium compositions: KSFM and embryonic fibroblast medium (EFM) mixed at a ratio of 1:1 with 5% (vol/vol) FBS [3] or KSFM and progenitor cell medium in a 1:1 ratio [5].

The search for optimal UDSC culture medium was described by Zhou et al. The authors cultivated primary cells in a medium that contained 10% (vol/vol) FBS to increase the initial cell adherence and survival. Later, for cellular expansion, authors cultivated cells in two distinct media: (a) RE (renal epithelial) proliferation medium [6] and (b) mixture of RE/MC (renal epithelial/mesenchymal cell) proliferation medium in a ratio 1:1. According to the results, UDSC grew in both media, but the proliferation rate was higher in the latter [7].

Urothelial cells grow in serum-free medium, which is a different feature from UDSC and BMSC [3,8]. Another experimental condition that distinguishes urothelial cells from UDSC and MSC is the need for collagen matrices and salt concentration. To improve cellular growth and the formation of stratified epithelium, it is required to cultivate urothelial cells in a medium with high concentrations of calcium chloride [9,10] on flasks covered by collagen [11,12]. Moreover, UDSC long-term cultivation was more efficient by cultivating cells on porous collagen matrix in a cell medium supplemented with 5% FBS, hormones and calcium [10,13]. Instead of collagen matrices, some researchers seeded cells on feeder cells (embryonic mesenchymal-derived (Swiss 3T3)) [11], at least for the primary culture [14,15]. In comparison to urothelial cells, UDSC do not require such specific conditions.

Whenever urothelial differentiation is required, UDSC can be cultivated on plates, covered by collagen, in a serum-free keratinocyte medium [16] or in a mixture of medium containing equal amounts of KSFM and EFM with 2% FBS [17]. EGF is a growth factor required for urothelial differentiation [3,17]. Urothelial differentiation of cells is characterized by specific cobblestone morphology [18]; the presence of tight junction genes and protein markers (ZO-1 and E-cadherin) and the expression of uroplakin Ia and III, AE1/AE3 and CK-7 [3]; pancytokeratins and cytokeratin20 [16]. Stratification of confluent UDSC monolayer cultures can be induced in a culture medium containing 1.5 mM calcium chloride [19].

Addition of EGF may also induce endothelial differentiation of UDSC, as it was demonstrated by Dong et al. [5]. In comparison to the previous urothelial differentiation, cells were cultivated in a mixture of DMEM 10% FBS with KSFM at a 1:4 ratio and 30 ng/mL EGF. A more specific medium content was later used by Bharadwaj S. et al. [17]. In this work, cells were cultured on fibronectin-coated plates in an endothelial basal medium (EBM2, Lonza) supplemented with 50 ng/mL VEGF. Such medium was used also for the induction of MSC endothelial differentiation [20]. This differentiation was demonstrated through measuring endothelial cell-specific gene transcripts (vWF and CD31); protein markers (CD31 [5], vWF, KDR, FLT1

and eNOS) and a tight endothelial junction marker (VE-cadherin) [17]. Formation of vessel-like structures was also visualized during cultivation on Matrigel [17].

For the induction of UDSC neuronal differentiation, Bhadawaraj et al. described a specific protocol. Cells were initially grown in pre-induction medium containing DMEM with 20% FBS and 10 ng/mL basic fibroblast growth factor (bFGF). Then, 24 hours later, the medium was replaced with a nerve induction medium for a further 48 hours. As a result, UDSC exhibited several neurogenic extensions and processes; approximately 40% of cells expressed the neuronal specific protein markers nestin, S100, NF200 and glial fibrillary acidic protein [17]. In other studies, the neural differentiation medium consisted of DMEM/Hamm's F12 medium supplemented with 20 ng/mL EGF, 40 ng/mL bFGF, 2% B27, 1% nonessential amino acid, 1% l-glutamine and 1% insulin-transferrin-selenite. As a result of the neurogenic differentiation of UDSC, an increased number of Sox2- and Nestin-positive cells was confirmed by the significantly increased gene expression of neuronal progenitor cell markers Nestin and Sox2, while mature neuron marker β-III-tubulin was not upregulated [21]. Alternatively, neurogenic differentiation of UDSC was also induced by cultivation in a commercial NeuroCult NS-A differentiation kit (StemCell) that lead to neuron-specific morphological changes and β-III-tubulin gene expression [18].

Confirming the initial ideas that UDSC have mesodermal origin, these cells can be differentiated into derivatives of mesoderm—osteogenic, chondrogenic, myogenic and adipogenic differentiation lineages [22].

Differentiation of UDSC into smooth muscle cells can be performed by cultivation in DMEM (high glucose) and EFM supplemented with 2.5 ng/mL transforming growth factor beta 1 (TGFb1), 5.0 ng/mL platelet-derived growth factor-BB (PDGF-BB) and 10% FBS [3,5,17]. Confirming the characteristics of smooth muscle, cells have elongated and spindle-shaped morphology, expression of desmin [3,5], major histocompatibility complex (MHC), alpha-smooth muscle actin (α-SMA), vimentin and in vitro contractility on collagen lattices [3,17].

For osteogenic differentiation, most publications reported the use of osteogenic induction media from commercial sources [18,21]. During cultivation, 70%–80% of UDSCs appeared to produce mineralized tissue that was confirmed by von Kossa, alkaline phosphatase and Alizarin Red S staining for calcium deposition. Adipogenic commercial medium is widely used to demonstrate adipogenic differentiation of UDSC [18,21]. After adipogenic induction, cells were positive for oil red-O staining and 30%–40% expressed adipocyte gene markers—peroxisome proliferator-activated receptor, acetyl-CoA synthase, adiponectin, CCAAT/enhancer-binding protein a, fatty acid binding protein 4 and lipoprotein lipase [17,21,23]. For chondrogenic differentiation, UDSC can be cultivated with commercial chondrogenic induction medium,

[18,21] and cells can be stained with Alcian blue, toluidine blue, and safranin-O for labeling sulfated glycosaminoglycan proteins to check the efficiency of differentiation. According to the authors' results obtained, about 60% of the induced UDSCs expressed the chondrogenic lineage markers Sox9, collagen-II and aggrecan [21]. However, in comparison to osteo- and myogenic differentiation, chondrogenic differentiation of UDSC was less efficient [2,17].

UDSC may also be differentiated into skeletal muscle cells. Such differentiation may be induced by two methods: (a) DMEM with an addition of 50 μM hydrocortisone, 0.1 μM dexamethasone, 10% FBS and 5% horse serum [17] and (b) conditioned medium from skeletal muscle cell cultures for 12 hours [18,24]. After one month of cultivation, UDSC displayed an elongated spindle-shaped morphology and expression of skeletal muscle-related transcripts (MyoD and myogenin). Moreover, 50%–60% of cells stained for MyoD formed myotube-like structures [17]. It was demonstrated that this myogenic differentiation properties of UDSC and ADCS were comparable and that the expression of MyoD in UDSC cultures was higher. Thus, UDSC seem to be more prone to a skeletal muscle differentiation lineage commitment [18].

Therefore, UDSC medium content and the presence of different supplements play a crucial role in cell viability, growth and phenotype. The culture medium used may contribute to distinguishing various cell types and to induce differentiation into multiple cell lineages, allowing for a high versality of this type of cell for multiple purposes.

References

1. Zhang, Y.Y.; Ludwikowski, B.; Hurst, R.; Frey, P. Expansion and long-term culture of differentiated normal rat urothelial cells in vitro. *In Vitro Cell Dev Biol Anim* **2001**, *37*, 419-429, doi:10.1290/1071-2690(2001)037<0419:EALTCO>2.0.CO;2.
2. Zhang, Y.Y.; Frey, P. Growth of cultured human urothelial cells into stratified urothelial sheet suitable for autografts. *Adv Exp Med Biol* **2003**, *539*, 907-920.
3. Bharadwaj, S.; Liu, G.; Shi, Y.; Markert, C.; Andersson, K.E.; Atala, A.; Zhang, Y. Characterization of urine-derived stem cells obtained from upper urinary tract for use in cell-based urological tissue engineering. *Tissue Eng Part A* **2011**, *17*, 2123-2132, doi:10.1089/ten.TEA.2010.0637.
4. Liu, G.; Wang, X.; Sun, X.; Deng, C.; Atala, A.; Zhang, Y. The effect of urine-derived stem cells expressing VEGF loaded in collagen hydrogels on myogenesis and innervation following after subcutaneous implantation in nude mice. *Biomaterials* **2013**, *34*, 8617-8629, doi:10.1016/j.biomaterials.2013.07.077.
5. Dong, X.; Zhang, T.; Liu, Q.; Zhu, J.; Zhao, J.; Li, J.; Sun, B.; Ding, G.; Hu, X.; Yang, Z., et al. Beneficial effects of urine-derived stem cells on fibrosis and apoptosis of myocardial, glomerular and bladder cells. *Mol Cell Endocrinol* **2016**, *427*, 21-32, doi:10.1016/j.mce.2016.03.001.

6. Zhou, T.; Benda, C.; Duzinger, S.; Huang, Y.; Li, X.; Li, Y.; Guo, X.; Cao, G.; Chen, S.; Hao, L., et al. Generation of induced pluripotent stem cells from urine. *J Am Soc Nephrol* **2011**, *22*, 1221-1228, doi:10.1681/ASN.2011010106.
7. Zhou, T.; Benda, C.; Dunzinger, S.; Huang, Y.; Ho, J.C.; Yang, J.; Wang, Y.; Zhang, Y.; Zhuang, Q.; Li, Y., et al. Generation of human induced pluripotent stem cells from urine samples. *Nat Protoc* **2012**, *7*, 2080-2089, doi:10.1038/nprot.2012.115.
8. Hintz, D.S.; Sens, M.A.; Jenkins, M.Q.; Sens, D.A. Tissue culture of epithelial cells from urine. I. Serum-free growth of cells from newborn infants. *Pediatr Pathol* **1984**, *2*, 153-163.
9. Reznikoff, C.A.; Loretz, L.J.; Pesciotta, D.M.; Oberley, T.D.; Ignjatovic, M.M. Growth kinetics and differentiation in vitro of normal human uroepithelial cells on collagen gel substrates in defined medium. *J Cell Physiol* **1987**, *131*, 285-301, doi:10.1002/jcp.1041310302.
10. Southgate, J.; Hutton, K.A.; Thomas, D.F.; Trejdosiewicz, L.K. Normal human urothelial cells in vitro: proliferation and induction of stratification. *Lab Invest* **1994**, *71*, 583-594.
11. Howlett, A.R.; Hodges, G.M.; Rowlatt, C. Epithelial-stromal interactions in the adult bladder: urothelial growth, differentiation, and maturation on culture facsimiles of bladder stroma. *Dev Biol* **1986**, *118*, 403-415.
12. Ludwikowski, B.; Zhang, Y.Y.; Frey, P. The long-term culture of porcine urothelial cells and induction of urothelial stratification. *BJU Int* **1999**, *84*, 507-514.
13. Chlapowski, F.J. Long term growth and maintenance of stratified rat urothelium in vitro. *Cell Tissue Kinet* **1989**, *22*, 245-257.
14. Fossum, M.; Gustafson, C.J.; Nordenskjold, A.; Kratz, G. Isolation and in vitro cultivation of human urothelial cells from bladder washings of adult patients and children. *Scand J Plast Reconstr Surg Hand Surg* **2003**, *37*, 41-45.
15. Fossum, M.; Lundberg, F.; Holmberg, K.; Schoumans, J.; Kratz, G.; Nordenskjold, A. Long-term culture of human urothelial cells--a qualitative analysis. *Cells Tissues Organs* **2005**, *181*, 11-22, doi:10.1159/000089965.
16. Nagele, U.; Maurer, S.; Feil, G.; Bock, C.; Krug, J.; Sievert, K.D.; Stenzl, A. In vitro investigations of tissue-engineered multilayered urothelium established from bladder washings. *Eur Urol* **2008**, *54*, 1414-1422, doi:10.1016/j.eururo.2008.01.072.
17. Bharadwaj, S.; Liu, G.; Shi, Y.; Wu, R.; Yang, B.; He, T.; Fan, Y.; Lu, X.; Zhou, X.; Liu, H., et al. Multipotential differentiation of human urine-derived stem cells: potential for therapeutic applications in urology. *Stem Cells* **2013**, *31*, 1840-1856, doi:10.1002/stem.1424.
18. Kang, H.S.; Choi, S.H.; Kim, B.S.; Choi, J.Y.; Park, G.B.; Kwon, T.G.; Chun, S.Y. Advanced Properties of Urine Derived Stem Cells Compared to Adipose Tissue Derived Stem Cells in Terms of Cell Proliferation, Immune Modulation and Multi Differentiation. *Journal of Korean medical science* **2015**, *30*, 1764-1776, doi:10.3346/jkms.2015.30.12.1764.
19. Li, J.; Luo, H.; Dong, X.; Liu, Q.; Wu, C.; Zhang, T.; Hu, X.; Zhang, Y.; Song, B.; Li, L. Therapeutic effect of urine-derived stem cells for protamine/lipopolysaccharide-induced interstitial cystitis in a rat model. *Stem Cell Res Ther* **2017**, *8*, 107, doi:10.1186/s13287-017-0547-9.
20. Oswald, J.; Boxberger, S.; Jorgensen, B.; Feldmann, S.; Ehninger, G.; Bornhauser, M.; Werner, C. Mesenchymal stem cells can be differentiated into endothelial cells in vitro. *Stem Cells* **2004**, *22*, 377-384, doi:10.1634/stemcells.22-3-377.
21. Guan, J.J.; Niu, X.; Gong, F.X.; Hu, B.; Guo, S.C.; Lou, Y.L.; Zhang, C.Q.; Deng, Z.F.; Wang, Y. Biological characteristics of human-urine-derived stem cells: potential for cell-based therapy in neurology. *Tissue Eng Part A* **2014**, *20*, 1794-1806, doi:10.1089/ten.TEA.2013.0584.
22. Dominici, M.; Le Blanc, K.; Mueller, I.; Slaper-Cortenbach, I.; Marini, F.; Krause, D.; Deans, R.; Keating, A.; Prockop, D.; Horwitz, E. Minimal criteria for defining multipotent mesenchymal

stromal cells. The International Society for Cellular Therapy position statement. *Cytotherapy* **2006**, *8*, 315-317, doi:10.1080/14653240600855905.
23. Guan, J.; Zhang, J.; Li, H.; Zhu, Z.; Guo, S.; Niu, X.; Wang, Y.; Zhang, C. Human Urine Derived Stem Cells in Combination with beta-TCP Can Be Applied for Bone Regeneration. *PLoS One* **2015**, *10*, e0125253, doi:10.1371/journal.pone.0125253.
24. Adamowicz, J.; Kloskowski, T.; Tworkiewicz, J.; Pokrywczynska, M.; Drewa, T. Urine is a highly cytotoxic agent: does it influence stem cell therapies in urology? *Transplant Proc* **2012**, *44*, 1439-1441, doi:10.1016/j.transproceed.2012.01.128.
